# Association between sugar consumption, sociodemographic, anthropometric and biochemical profiles

**DOI:** 10.4102/phcfm.v5i1.546

**Published:** 2013-11-27

**Authors:** Zorada Hattingh, Catharina J. Bester, Corinna M. Walsh

**Affiliations:** 1 Faculty of Management Sciences, Central University of Technology, South Africa; 2 Department of Biostatistics, Faculty of Health Sciences, University of the Free State, South Africa; 3 Department of Nutrition and Dietetics, Faculty of Health Sciences, University of the Free State, South Africa

## Abstract

**Background:**

The increase in prevalence of coronary heart disease, type 2 diabetes, obesity and abnormal blood lipid levels has raised the question of a possible relationship between these conditions and the consumption of sugar.

**Objectives:**

This study investigated the sugar consumption of financially-restricted Black women in Mangaung, South Africa.

**Method:**

Five hundred women were selected randomly and divided into younger (25–34 years) and older (35–44 years) groups. Dietary intake, sociodemographic status, anthropometry and biochemical data were obtained. Total sugar (TS) and added sugar (AS) consumption were compared between older and younger women as well as sociodemographic, anthropometric and biochemical categories.

**Results:**

AS intake contributed 12% and 13% of total energy intake in younger and older women, respectively. AS consumption was higher in younger women living in brick houses and those who possessed a microwave oven. In older women, it was higher in husband-headed households. Underweight women with the lowest body mass index had higher sugar consumption than overweight and/or obese women. Women with a lower body fat percentage had a higher AS consumption than women with a high body fat percentage. Sugar consumption was significantly lower in younger women with elevated serum lymphocyte counts. TS and AS consumption was higher in younger women with elevated serum glucose levels. Older women with elevated serum insulin had a significantly higher TS consumption compared to those with normal insulin concentrations.

**Conclusion:**

The amounts of TS and AS consumed by women in this observational study were unlikely to contribute to overweight and/or obesity.

## Abstrait

**Association entre consommation de sucre et profils sociodémographiques, anthropométriques et biochimiques**

**Contexte:**

La prévalence croissante de la cardiopathie coronarienne, du diabète de type 2, de l'obésité et de taux anormaux de lipides a soulevé la question d'une relation possible entre ces maladies et la consommation de sucre.

**Objectifs:**

Cette étude a enquêté sur la consommation de sucre des femmes noires financièrement limitées à Mangaung en Afrique du Sud.

**Méthode:**

Un groupe de cinq cents femmes ont été sélectionnées aléatoirement et divisé en deux groupes: les 25-34 ans et les 35-44 ans. Des données sur la consommation alimentaire, le statut sociodémographique, l'anthropométrie et la biochimie ont été recueillies. La consommation de Sucres Totaux (ST) et de Sucres Ajoutés (SA), les catégories sociodémographiques, anthropométriques et biochimiques ont été comparées entre les deux groupes de femmes.

**Résultats:**

L'apport de SA représentait respectivement 12% et 13% de l'apport énergétique total des femmes jeunes et âgées. La consommation de SA était supérieure chez les jeunes femmes vivant dans des maisons en briques et chez celles possédant un four à micro-onde. Chez les femmes âgées, elle était supérieure dans les foyers où l'homme était le chef de famille. Les femmes maigres présentant l »indice de masse corporelle le plus faible avaient une consommation de sucre supérieure à celle des femmes en surpoids et/ou obèses. Les femmes présentant le taux de graisse corporelle le plus faible avaient une consommation de SA plus élevée que les femmes à taux de graisse corporelle élevé. La consommation de sucre était bien plus basse chez les jeunes femmes présentant une numération des lymphocytes sériques élevée. La consommation en ST et SA était supérieure chez les jeunes femmes présentant un taux de glucose sérique élevé. Les femmes âgées présentant un taux sérique d'insuline élevé consommaient bien plus de ST que celles présentant des concentrations normales d'insuline.

**Conclusion:**

Il est improbable que les quantités de ST et de SA consommées par les femmes de cette étude d'observation aient contribué au surpoids et/ou à l'obésité.

## Introduction

Remarkable changes in the food supply system, steered by modern technology, have been paving the way to an adjustment in the food consumption patterns of numerous populations.^1^ As such, the global access to processed foods which are particularly high in fat^2^ and sugar, has been drawing much attention.^2, 3, 4, 5^ In addition, the adverse health consequences of increasing levels of urban exposure merging with the nutrition transition have been scrutinised by both international^1, 2, 6^ and South African researchers.^7, 8, 9, 10, 11^

When consumed primarily as whole grains, fruit and vegetables, carbohydrates not only provide the body with energy and micronutrients, but also reduce the risk of becoming overweight and obese.^12^ However, carbohydrates that are provided from these foods are much more nutrient dense than those provided by added sugar (AS). Although an upper limit for sugar intake has not been set, a maximum of 25% or less of daily energy intake from AS has been suggested.^13^ The World Health Organization (WHO)^14^ advises an intake of AS not exceeding 10% of the total daily energy intake. In Western countries,^5, 15^ AS consumption exceeds the recommended guidelines. In this regard, the significant increase in the consumption of sugar-sweetened beverages overloaded with high-fructose corn syrups,^16, 17, 18^ which are also added to several other manufactured products, has come under scrutiny.^18, 19^ Although sucrose and not high-fructose corn syrup is used to sweeten beverages and other foods in South Africa, both sugar and high-fructose corn syrup are more or less equal mixes of fructose and glucose molecules (covalently bonded into a disaccharide in the case of sucrose and a mix of free fructose and glucose in the case of high-fructose corn syrup), so that metabolically there should be no difference in their effect on the body.

Limited data on the total sugar (TS) and AS consumption of urban Black South Africans have been published. Urban Black adults from the Cape Peninsula, South Africa, have been shown to consume 47–52 g per day of AS,^20^ whilst urban Black South African women habitually consume a mean of 94.2 grams of sugar per day, in contrast to their rural counterparts’ intake of 44.6 g per day.^21^ The South African Food Based Dietary Guideline, ‘Eat and drink food and drinks that contain sugar sparingly’,^22^ implies that within a diet supplying at least 55% of the daily energy intake in the form of various carbohydrate-rich foods, some room for the intake of sugar is allowed.^23^

The global increase in the prevalence of coronary heart disease, type 2 diabetes, obesity and abnormal blood lipid levels, has prompted the question of a possible relationship between these conditions and the consumption of AS.^24^

Whilst some authors contend that there is no substantial evidence that an increased intake of AS contributes to an increase in body mass index (BMI) or obesity,^17, 25^ others argue that the effect of sugar on obesity and other metabolic disorders needs to be explored further.^3^

This paper documents the possible link between TS and AS consumption, fat consumption, sociodemographic and anthropometric status and selected biochemical variables of urbanised Black women in Mangaung, in the Free State Province of South Africa.

## Research method and design

For this cross-sectional study initiated in 2000, a representative sample of 500 anti-retroviral (ARV) naïve, non-pregnant, pre-menopausal Black women were selected randomly. Women were divided into two age groups, namely 25–34 years and 35–44 years, in order to enable the researchers to compare the study results with a similar study conducted within the same geographical area.^33^ Township maps from two formal settlements and two informal settlements in Mangaung were used to select the sample. These settlements were considered as being representative of the township. The residential plots in the four areas were counted and numbered. An explanation of the random selection of residences and possible participants by a trained community healthcare worker has been published elsewhere.^26^

The structured questionnaire employed to establish the sociodemographic status of the women included questions on the number of years residing in an urban area, smoking habits, household composition, marital status, highest level of education, employment status of the respondent or husband and/or partner, head of the household, household facilities and type and size of dwelling. The questionnaire was administered during a structured interview with each woman, with Sesotho and isiXhosa interpreters assisting the researchers. Interviews were conducted at the research centre at the Central University of Technology, Free State. A validated, culture-sensitive Quantitative Food Frequency Questionnaire (QFFQ), developed for the Transition and Health during Urbanisation of South Africans (THUSA) study conducted by the University of North West in South Africa, was used in order to determine dietary intake.^27^ The questionnaire included traditional and Western foods and local foods commonly sold in the Mangaung area by food vendors. A detailed description of the methodology employed to determine dietary intake of energy and macronutrients has been published in a separate paper.^28^

For the purpose of this study, TS consumption included foods containing sugar in its natural form and sugar added to foods and beverages. AS refers to sugar added to porridge or cereal, sweet spreads such as jam, honey or syrup used on bread, sugar added during the preparation of vegetables and sugar and condensed milk added to tea or coffee, fizzy drinks, snacks, desserts, sweets, biscuits, cakes, tarts, custard and condiments such as tomato sauce and chutney.

Standardised methods (of which the procedures have been described in separate publications from this study) were used to determine BMI,^29^ body fat percentage^30^ and biochemical values, applying calibration and quality control specimens as supplied by the manufacturers of the respective methods for HIV and other selected biochemical variables.^31^

### Statistical analyses

Data were processed using SAS software.^32^ All data sets were categorised into two age groups, 25–34 years (younger women) and 35–44 years (older women). For each age group, continuous variables were described by means and standard deviations or medians and percentiles, as applicable. Categorical variables were described by means of frequencies and percentages. Within each age group, median TS, AS and total fat intake were compared for categorical variables using the relevant Kruskall-Wallis or Wilcoxon test and by calculating the 95% confidence intervals (CIs) for the median unpaired difference.

## Results

Of the total sample of 500 women recruited, 488 met the inclusion criteria. Of the 12 women who did not qualify to participate, four women were found to be pregnant when examined by a medical practitioner and eight did not meet the age requirement. Two hundred and seventy-three (55.9%) of the women were 25–34 years of age and 215 (44.1%) were 35–44 years of age. In the 25–34 years group, 167 (61.1%) women were HIV-infected and 82 (38.1%) in the 35–44 years group. Although most women were not aware of their HIV status and therefore not taking anti-retroviral medication at the time of the study, they gave informed consent to have their HIV status determined. The median energy intake was high, ranging from 10 656 kJ per day in the older group to 11 507 kJ per day in the younger group. When macronutrient intake was expressed as a percentage of total energy (TE) intake, the distribution was very similar in both age groups, with carbohydrates contributing between 51% and 53%, protein 12% and fat between 31% and 32%. The median TS showed an intake of 84 g per day in both age groups. The median AS consumption was 75 g per day for younger women and 69 g per day for older women, contributing 12% and 13% of the total daily energy intake in younger and older women, respectively. The median total fat intake was 99 g per day for younger and 89 g per day for older women.

The mean intake by mass of the 20 most frequently-consumed foods in the two age groups is presented in Tables 1 and 2 respectively. Foods and beverages are listed from 1 to 20 in sequence of popularity, with Number 1 in the Tables indicating the food most frequently consumed (in grams) by the total sample of women. Frequently-consumed foods were very similar for the two groups, with English tea, fresh and/or whole milk, maize porridge and coffee ranking highest on the list. White sugar and foods containing AS, such as fizzy drinks and cordials, appeared high on the list of popular foods consumed by women of both age groups.

**TABLE 1:** Total mean intake by mass of the 20 most frequently-consumed foods in the 25–34 year (*n* = 273) group.

Number	Food item	Total mean intake per day (grams)
1	English tea	93 539
2	Soft maize-meal porridge	88 344
3	Whole and/or fresh milk	64 288
4	Coffee	59 315
5	Stiff maize-meal porridge	42 116
6	Fizzy drinks	29 525
7	Rooibos tea	28 872
8	Beer	26 898
9	Brown bread	24 810
10	Mabella porridge	19 162
11	Cordials	12 753
12	White sugar	12 199
13	Apple	10 551
14	Banana	10 312
15	Samp and beans	9 378
16	Sorghum beer	8 996
17	White rice	7 835
18	White bread	7 645
19	Fresh fruit juice	7 143
20	Oranges	7 098

**TABLE 2:** Total mean intake by mass of the 20 most frequently-consumed foods in the 35–44 year (*n* = 215) group.

Number	Food item	Total mean intake per day (grams)
1	English tea	97 901
2	Whole and/or fresh milk	61 476
3	Soft maize-meal porridge	57 017
4	Stiff maize-meal porridge	50 592
5	Coffee	43 008
6	Beer	23 787
7	Rooibos tea	17 338
8	Mabella porridge	15 328
9	Brown bread	14 549
10	Fizzy drinks	13 332
11	White sugar	10641
12	Samp and beans	8 934
13	Cordials	7 629
14	White bread	7 520
15	Mageau and/or Motogo	7 386
16	Apple	7 230
17	Orange	6 252
18	Spinach cooked with onion and potato	6 132
19	Banana	5 839
20	Oats porridge	5 224

AS consumption in married and unmarried women was very similar, ranging between a median of 69 g per day for older women and a median intake of 76 g per day for younger women. Table 3 summarises the sugar and fat consumption of women in different sociodemographic categories. The fat intake of married women in both age groups tended to be higher than that of unmarried women. In the older group, women from households headed by a husband had a significantly higher intake of TS and AS (*p* = 0.028 and *p* = 0.022, respectively). AS consumption was significantly higher in younger women who lived in brick houses (*p* = 0.035). Younger women who possessed a microwave oven had significantly higher TS and AS intakes (*p* = 0.030 and *p* = 0.007, respectively) and total fat intake (*p* = 0.033). In the older group, the difference was significant for total fat intake (*p* = 0.030).

**TABLE 3:** The association between sugar- and fat consumption and sociodemographic variables of women 25–34 and 35–44 years of age.

Sociodemographic variables	Age group							

	25–34 years (*n* = 273)				35–44 years (*n* = 215)			
	
	*n* (%)	Median intake (g)	*p*-value	95% CI for median unpaired difference	*n* (%)	Median intake (g)	*p*-value	95% CI for median unpaired difference
**Total sugar**								
Don't smoke Smoke	183 90	85 82	0.338	-3.9; 15.3	91 124	89 78	0.262	-3.3; 17.1
**Added sugar**								
Don't smoke Smoke	183 90	76 69	0.181	-2.1; 17.4	91 124	69 68	0.622	-6.4; 12.0
**Total fat**								
Don't smoke Smoke	183 90	101 93	0.406	-5.4; 15.4	91 124	102 81	0.083	0.4; 22.0
**Total sugar**								
Married Unmarried	52 221	86 83	-0.365; 20.0	-5.7; 20.0	49 166	98 79	0.180	-2.16; 21.7
**Added sugar**								
Married Unmarried	52 221	76 73	0.799	-10.3; 14.7	49 166	69 69	0.533	-7.1; 14.8
**Total fat**								
Married Unmarried	52 221	108 97	0.170	-2.5; 24.5	49 166	93 88	0.241	-3.2; 22.2
**Total sugar**								
Husband Other	77 196	79 85	0.335	-16.2; 4.8	75 140	98 77	0.028*	3.7; 24.8*
**Added sugar**								
Husband Other	77 196	71 76	0.422	-15.6; 5.5	75 140	76 63	0.022*	4.0; 24.2*
**Total fat**								
Husband Other	77 196	92 100	0.390	-17.2; 5.3	75 140	94 83	0.138	-1.0; 21.5
**Total sugar**								
Brick Other	200 73	87 74	0.086	0.4; 20.8*	158 57	88 74	0.032*	3.2; 25.1*
**Added sugar**								
Brick Other	200 73	79 61	0.035*	3.0; 23.1*	158 57	71 58	0.097	0.1; 20.0*
**Total fat**								
Brick Other	200 73	102 84	0.011	5.7; 27.3*	158 57	94 73	0.045	2.3; 27.1*
**Total sugar**								
Yes No	30 243	106 81	0.030*	5.5; 40.8*	15 200	91 83	0.086	0.7; 46.6*
**Added sugar**								
Yes No	30 243	105 71	0.007*	11.0; 44.2*	15 200	73 69	0.180	-2.3; 33.6
**Total fat**								
Yes	30 243	120 97	0.033*	4.8; 39.7*	15 200	118 86	0.030*	7.3; 49.1*
								

CI, confidence interval

* Statistically-significant median unpaired difference: *p*-value <0.05.

Table 4 depicts the sugar and fat consumption of younger and older women and their respective anthropometric status categories. More than 90% of all women had a body fat percentage in the fat and overweight category. Both TS and AS consumption was higher in women with the lowest BMI, and as BMI increased, sugar consumption decreased. Women with the highest body fat percentage also had the lowest intake of both TS and AS.

**TABLE 4:** The association between sugar- and fat consumption and anthropometric variables of women 25–34 and 35–44 years of age.

Variable	Age group							

	25–34 years			Category comparison:95% CI for median unpaired difference	35–44 years			Category comparison: 95% CI for median unpaired difference
	
	*n*	Median intake (g)	*p-value*		*n*	Median intake (g)	*p-value*	
**BMI *(kg/m^2^)*TS**	**272**	-	-	-	**215**	-	-	-
< 18.5 18.5–24.9 ≥ 25	7 121 144	107 90 79	0.144	1–2: -27.9; 65.6 1–3: -19.5; 76.6 2–3: 1.4; 20.2*	9 95 111	81 88 80	0.830	2–3: -8.8; 12.3
**AS**								
< 18.5 18.5–24.9 ≥ 25	7 121 144	94 82 68	0.202	1–2: -26.8; 42.3 1–3: -17.8; 52.2 2–3: 0.22; 19.2*	9 95 111	85 73 63	0.428	2–3: -1.7; 17.1
**Total fat**								
< 18.5 18.5–24.9 ≥ 25	7 121 144	119 103 93	0.095	1–2: -35.9; 56.3 1–3: -21.2; 66.6 2–3: 2.9; 23.2*	9 95 111	96 90 84	0.976	2-3: -9.4; 11.8
**%of body fat TS**	**273**	-		-	**215**	-	-	-
< 15% 15–22% 23–26% 27–32% > 32%	1 8 24 46 194	228 95 85 89 80	0.304	4–5: -2.1; 21.4	0 3 13 30 169	- 68 89 86 80	0.902	4–5: -10.1; 18.5
**AS**								
< 15% 15–22% 23–26% 27–32% > 32%	1 8 24 46 194	94 75 94 82 70	0.608	4–5: -3.2; 21.7	0 3 13 30 169	- 91 93 73 65	0.693	4–5: -6.9; 20.1
**Total fat**								
< 15% 15–22% 23–26% 27–32% > 32%	1 8 24 46 194	216 105 87 105 98	0.300	4–5: -3.3; 22.9	0 3 13 30 169	- 111 94 76 89	0.756	4–5: -20.1; 10.8

BMI, body mass index; TS, total sugar; AS, added sugar

* Statistically-significant median unpaired difference: *p*-value < 0.05.

Table 5 displays the association between the consumption of TS, AS and total fat and selected fasting biochemical variables. Younger women with elevated total lymphocyte counts consumed significantly less sugar than those with normal lymphocyte counts (*p* = 0.041 for TS and *p* = 0.013 for AS).

**TABLE 5:** The association between sugar- and fat intake and biochemical variables of women 25–34 years and 35–44 years of age.

Biochemical variables	Age group							

	25–34 years				35–44 years			
	
	*n*	Median intake (g)	*p-*value	Category comparison: 95% CI for median unpaired difference	*n*	Median intake (g)	*p-*value	Category comparison: 95% CI for median unpaired difference
**Lymphocytes TS**	**262**	-	-	-	**210**	-	-	-
< 0.8 x 109/L 0.8–3.3 x 109/L > 3.3 x 109/L	0 239 23	- 86 71	0.041*	2–3: 4.2; 35.1*	2 196 12	94 84 102	0.995	2–3: -23.7; 22.4
**AS**								
< 0.8 x 109/L 0.8–3.3 x 109/L > 3.3 x 109/L	0 239 23	- 77 49	0.013*	2–3: 7.8; 38.4*	2 196 12	62 69 63	0.903	2–3: -17.9; 23.9
**Total fat**								
< 0.8 x 109/L 0.8–3.3 x 109/L > 3.3 x 109/L	0 239 23	- 99 100	0.477	2-3: -9.2; 25.0	2 196 12	103 89 100	0.712	2–3: -29.5; 12.6
**Total proteins TS**	**273**	-	-	-	**214**	-	-	-
< 60 g/L 60–82 g/L > 82 g/L	0 88 185	- 77 86	0.092	2–3: -19.7; -0.2	0 88 126	80 85	0.942	2–3: -10.7; 9.5
**AS**								
< 60 g/L 60–82 g/L > 82 g/L	0 88 185	- 70 76	0.669	2–3: -12.9; 7.9	0 88 126	- 63 73	0.624	2–3: -11.9; 6.3
**Total fat**								
< 60 g/L 60–82 g/L > 82 g/L	0 88 185	- 95 101	0.146	2–3: -19.3; 1.2	0 88 126	- 88 89	0.492	2–3: -6.2; 15.2
**Serum albumin TS**	**273**	-	-	-	**213**	-	-	-
< 34 g/L 34–48 g/L ≥ 48 g/L	14 224 35	85 82 95	0.113	2–3: -31.7; -3.6*	11 195 7	74 82 106	0.160	2-3: -47.7; 0.8
**AS**								
< 34 g/L 34–48 g/L ≥ 48 g/L	14 224 35	76 73 81	0.835	2–3: -18.9; 9.9	11 195 7	58 68 75	0.634	2–3: -26.3; 18.9
**Total fat**								
< 34 g/L 34–48 g/L ≥ 48 g/L	14 224 35	92 95 124	0.0003*	2–3: -54.7; -22.3*	11 195 7	76 89 107	0.654	2–3: -36.8; 12.1
Fibrinogen TS	257	-	-	-	203	-	-	-
< 1.5 g/L 1.5–4 g/L > 4 g/L	3 199 55	62 81 84	0.525	2–3: -13.6; 8.6	2 151 50	98 81 90	0.417	2–3: -20.7; 2.6
AS								
< 1.5 g/L 1.5–4 g/L > 4 g/L	3 199 55	48 73 73	0.302	2–3: -14.5; 8.8	2 151 50	55 68 70	0.558	2–3: -18.6; 4.8
Total fat								
< 1.5 g/L 1.5–4 g/L > 4 g/L	3 199 55	103 95 98	0.961	2–3: -13.9; 10.4	2 151 50	98 92 88	0.682	2–3: -6.3; 17.7
Serum glucose TS	272	-	-	-	215	-	-	-
< 3.05 mmol/L 3.05–6.38 mmol/L > 6.38 mmol/L	15 228 29	78 82 109	0.012*	1–2: -27.1; 6.6 1–3: -63.9; -14.6* 2–3: -44.1; -10.4*	13 193 9	97 81 89	0.431	1–2: -5.8; 35.2 1–3:-3.7; 51.2 2–3: -17.4; 32.7
**AS**								
< 3.05 mmol/L 3.05–6.38 mmol/L > 6.38 mmol/L	15 228 29	57 73 91	0.049*	1–2: -38.6; -2.3* 1–3: -49.1; -16.7* 2–3: -27.1; 4.6	13 193 9	98 69 61	0.058	1–2: 2.9; 46.1*
1–3: 8.7; 86.6*								
2–3: -2.1; 41.2								
Total fat								
< 3.05 mmol/L 3.05–6.38 mmol/L > 6.38 mmol/L	15 228 29	70 96 125	< 0.001*	1–2: -36.8; 4.9 1–3: -84.0; -30.2* 2–3: -60.0; -25.1*	13 193 9	116 87 87	0.142	1–2: 4.2; 43.1* 1–3: -1.9; 55.8 2–3: -23.1; 28.5
Serum insulin TS	272	-	-	-	215	-	-	-
< 2 µU/ml 2–25 µU/ml > 25 µU/ml	38 202 32	88 83 81	0.655	2–3: -9.4; 19.7	40 152 23	86 79 122	0.039*	2–3: -52.9; -12.6*
AS								
< 2 µU/ml 2–25 µU/ml > 25 µU/ml	38 202 32	76 75 60	0.619	2–3: -7.1; 21.7	40 152 23	63 70 76	0.372	2–3: -33.3; 3.3
Total fat								
< 2 µU/ml 2–25 µU/ml > 25 µU/ml	38 202 32	87 99 101	0.716	2–3: -7.7; 21.2	40 152 23	79 87 111	0.494	2–3: -30.1; 4.7
Triglycerides	273	-	-	-	214	-	-	-
TS								
< 2 mmol/L ≥ 2 mmol/L	257 16	85 79	0.983	-26.6; 22.6	185 29	87 72	0.128	-1.2; 26.3
AS								
< 2 mmol/L ≥ 2 mmol/L	257 16	75 73	0.887	-25.9; 19.4	185 29	73 49	0.004*	8.8; 32.1*
Total fat								
< 2 mmol/L ≥ 2 mmol/L	257 16	99 107	0.230	-46.3; 6.1	185 29	92 78	0.660	-12.1; 20.5
Serum cholesterol TS	273	-	-	-	214	-	-	-
< 5.2 mmol/L 5.2-7.8 mmol/L ≥ 7.8 mmol/L	218 43 12	82 92 93	0.059	1–2&3: -17.7; 5.2	153 59 2	84 86 78	0.927	1–2&3:-9.8; 12.1
AS								
< 5.2 mmol/L 5.2-7.8 mmol/L ≥ 7.8 mmol/L	218 43 12	73 81 66	0.743	1–2&3: -13.16; 10.2	153 59 2	68 71 41	0.428	1–2&3:-7.6; 12.5
Total fat								
< 5.2 mmol/L 5.2 < 7.8 mmol/L ≥ 7.8 mmol/L	218 43 12	95 120 118	0.008*	1–2&3: -36.6; 11.2	153 59 2	92 87 96	0.976	1–-2&3:-11.1; 11.3
HIV status TS								
HIV-infected HIV-uninfected	167 106	85 79	0.22	-4.2; 18.4	82 133	86 80	0.79	-10.7; 13.7
AS								
HIV-infected HIV-uninfected	167 106	75 72	0.96	-11.0; 11.9	82 133	71 68	0.74	-9.4; 13.0
Total fat								
HIV-infected HIV-uninfected	167 106	101 94	0.06	-0.5; 23.6	82 133	85 90	0.35	-18.9; 6.2

TS, total sugar; AS, added sugar

* Statistically-significant median unpaired difference.

In the younger group, a significant difference in sugar and total fat consumption between women with low-, normal- and high levels of serum glucose was evident (*p* = 0.012, *p* = 0.049 and *p* < 0.001 for TS, AS and total fat, respectively). As blood glucose levels tended to increase, so did TS and AS consumption. The total fat intake of women with elevated glucose levels was also higher than those with low- or normal glucose levels. In the older group, a significant difference in TS consumption was found between women with normal and high insulin levels (95% CI -52.9; -12.6). In addition, the *p*-value (0.039) indicated an overall effect of TS intake on insulin levels. Furthermore, a significant difference in AS consumption between women with normal- and high triglyceride levels was evident (95% CI 8.8; 32.1). As triglycerides tended to increase, AS consumption decreased. The small number of older women with high triglycerides (*n* = 29) requires that these results be interpreted with caution.

## Ethical considerations

The Ethics Committee of the Faculty of Health Sciences, University of the Free State, approved the study (ETOVS No. 02/00). The women gave informed consent, participation in the study was voluntary and confidentiality was assured.

## Trustworthiness

Validity of the information collected was assured by ensuring that all information was directly related to the aims and objectives of the study and based on a sound literature review.

## Discussion

The main objective of this cross-sectional study was to investigate the sugar consumption patterns of women (25–44 years) residing in Mangaung in the Free State province of South Africa. In the mid-1990s, Mollentze et al.^33^ forecast that the rapid urbanisation and adoption of a western diet within this township might accelerate the prevalence of certain chronic diseases of lifestyle.

Considering the excessive rates of AS consumption noted in the scientific literature,^5, 15^ the median intake of TS and the median intake of AS were lower than figures reported for other urban Black South African women.^21^ The contribution of 12% and 13% of AS, respectively, to the total daily energy intake of women of the two age groups was lower than the proposed maximum intake level of 25% TE from AS,^13^ but higher than the 10% of TE proposed by the WHO.^14^

The debate between sugar intake and body weight is a long-standing one. In Australia where obesity increased three-fold between 1980 and 2003, the per capita consumption of refined sugar decreased by 23% in the same period.^25^ Analysing the mean intakes by mass of the 20 most frequently-consumed foods, it became clear that sugar intake was mostly represented by sweetened beverages, refined grains and, to a lesser degree, some fruits. It is worth mentioning that the median energy and carbohydrate intake in our larger study exceeded current recommendations, whilst median fibre intake was inadequate for all women as reported in a previous publication of our study.^28^ In both age groups, most of the carbohydrates ingested ranked relatively high on the glycaemic index list,^28^ seen as a determinant of higher body weight.^12^ More than half of all women were either overweight or obese and the majority demonstrated an unfavourably high body fat percentage, associated with low levels of physical activity.^34^ Our finding that both TS and AS consumption was higher in women with the lowest BMI is supported by other studies stating that high carbohydrate diets seem to be less detrimental, with little evidence of the adverse effect of sugar on body weight.^25^ As with BMI, women with the highest fat percentage also had the lowest intake of both TS and AS. When considering absolute fat and sugar intakes, our results do not support an inverse relationship between sugar consumption and fat consumption. Fat consumption was similar to sugar consumption, with fat intake increasing as sugar intake increased. It should be noted that a large percentage of women included in the study were found to be HIV-infected (which was likely to impact on BMI, fat percentage and biochemical parameters). However, no significant differences in the TS, AS and fat intake of HIV-infected and HIV-uninfected women were found.

Educational status and income have been associated independently with AS consumption, implying that low income groups with a poor educational level include more AS into their diet.^24^ Poverty and food insecurity are related to a diet deficient in fresh fruit and vegetables, lean meats and fish, but high in palatable fat and energy-dense food.^35^ Lower-cost foods such as refined carbohydrates, AS and added fats are preferred food choices of those with a lower socioeconomic background.^36^ In general, these energy-dense food sources have a lower satiety value and may lead to passive overeating and most probably weight gain.^37^ In view of the lower socioeconomic background of the urbanised women in our study, younger women living in brick houses and those who possessed a microwave oven consumed significantly more sugar and total fat compared with women residing in informal housing. In the older group, a significant difference was observed between TS and AS intake in households headed by a husband. Although the participating women failed to report on their weekly or monthly income and unreliable results were obtained,^26^ it is likely that these significant differences point toward a more substantial diet in women with a higher disposable income.

The focus of several original studies and review articles has been aimed at the relationship between sugar consumption and the metabolic syndrome. A recent review by Bray^38^ confirmed that the fructose found in refined foods and sugar-sweetened beverages appears to be responsible for most of the metabolic risk factors. An excessive consumption of nutritive sweetened beverages has been associated with an increased risk of type 2 diabetes and cardiovascular disease through an increase in body weight.^39^ Stanhope et al.^40^ showed that fructose-sweetened beverages promoted *de novo* lipid synthesis, dyslipidaemia, visceral adiposity and insulin resistance in overweight or obese adults in small-scale randomised controlled trials.

In two systematic reviews, one followed by a meta-analysis, Malik et al.^18, 41^ confirmed the link between an increase in sugar-sweetened beverage intake, weight gain and the metabolic syndrome. An analysis of American data collected between 1909 and 1997 established the positive relationship between the prevalence of type 2 diabetes and obesity, which proportionally increased with an increased refined carbohydrate intake.^19^ In the USA cohort Nurses’ Study II (1991–1999), weight gain and risk for type 2 diabetes were related positively with a regular consumption of sugar-sweetened beverages.^4^ Later data (1999–2006) revealed a correlation between the consumption of AS and blood lipid levels, with those adding more sugar to the diet being more prone to higher triglyceride levels and higher triglyceride:HDL cholesterol ratios.^42^ In our study, sugar consumption was not significantly different for women with low-, normal- or high levels of most blood measures, other than serum lymphocytes, glucose and insulin. Results from the 20 most frequently-consumed foods seem to indicate that the contribution of sugar-sweetened beverages (tea, fizzy drinks and cordials) toward sugar consumption was relatively high.

When reviewing short-term randomised controlled trials, Fried and Rao^43^ concluded that sucrose and fructose tended to increase serum triacylglycerol levels, but other dietary factors such as the total carbohydrate intake, types of dietary fat, carbohydrate and fibre need consideration. When reporting on longer-term studies, they found no long-term association between sugar or total carbohydrate intake and cardiovascular risk. However, these authors reported that results from the CARMEN randomised controlled trial that lasted six months showed that a high glycaemic-loaded diet was linked to higher serum triacylglycerol levels and an increased risk of coronary heart disease in women.^43^ In the current study, no significant differences between sugar intake, obesity or elevated total cholesterol levels were found. In the younger group, sugar consumption was significantly higher in women with increased serum glucose levels, whilst in the older group, both sugar and fat consumption increased as serum insulin concentrations increased, but the difference was not significant. When reviewing several studies, Daly^44^ concluded that despite the conflicting results regarding the role of sugar in glycaemic control and insulin sensitivity, extremely varying sucrose contents in the diet did not affect insulin sensitivity differently in overweight persons, which could also apply to our study.

Data collection of the dietary intake of individuals and groups remains complex and no method can be singled out as being the most perfect.^45^ In order to best ensure reliability and validity of the TE, TS and AS intake results in the present study, specially trained and skilled postgraduate interviewers interviewers in the field of Nutrition and Dietetics conducted the structured interviews. The participating women were instructed about the value of their contribution toward the study and a valid and reliable nutrient database was used to analyse data. However, the problem of over- or underreporting of real dietary intake remains a reality and has to be considered when interpreting our study results on the dietary intake of the selected food items.^46^ Although the results from the 20 most frequently-consumed foods seem to indicate that consumption of sugar from sugar-sweetened beverages was relatively high, the specific contribution of these drinks to sugar consumption was not determined.

## Conclusion

Although the median energy intake of all women was high, macronutrient distribution fell within the recommended limits, except for fat which was higher than the recommended maximum intake of 30% of TE. TS and AS intake contributed approximately 12% and 13% of TE in younger and older women, respectively. Sucrose-sweetened beverages such as tea, fizzy drinks and cordials were noted on the list of 20 most frequently-consumed foods. Sugar intake was the highest in both younger and older women residing in brick houses and in households that had a microwave oven. In older women, sugar intake was significantly higher in households headed by a husband.

Higher sugar consumption was associated with a lower BMI and fat percentage. However, an inverse relationship between reported absolute fat consumption and BMI was not found. Sugar consumption was not significantly different for women with low-, normal- or high levels of most blood measures, other than serum lymphocytes, glucose and insulin. Sugar consumption in the amounts ingested by the women included in this study did not have a negative impact on health as reflected by anthropometry and biochemical variables, indicating that there may be a threshold at which sugar consumption affects these parameters.

## Recommendations

To curb the existing health hazards in this township of being overweight and obese, more vigorous plans of action for lifestyle modification need to be put into effect in order to inspire these women to follow a healthy eating plan comprising foods with a high nutrient density. In combination with an easily-accessible community-based physical activity programme, the prevailing problem could be mitigated.
